# FOXP3 Inhibits the Metastasis of Breast Cancer by Downregulating the Expression of MTA1

**DOI:** 10.3389/fonc.2021.656190

**Published:** 2021-07-07

**Authors:** Chenlin Liu, Jun Han, Xiaoju Li, Tonglie Huang, Yuan Gao, Baolong Wang, Kuo Zhang, Shuning Wang, Wangqian Zhang, Weina Li, Qiang Hao, Meng Li, Yingqi Zhang, Cun Zhang

**Affiliations:** State Key Laboratory of Cancer Biology, Biotechnology Center, School of Pharmacy, The Fourth Military Medical University, Xi’an, China

**Keywords:** metastasis, breast cancer, FOXP3, MTA1, transcription factor

## Abstract

**Background:**

FOXP3, as a tumour suppressor gene, has a vital function in inhibiting the metastasis of breast cancer cells, but the mechanisms by which it inhibits metastasis have not been fully elucidated. This study intended to explore a new mechanism by which FOXP3 inhibits breast cancer metastasis.

**Methods:**

Bioinformatic analysis was performed to identify potential downstream molecules of FOXP3. The function of FOXP3 in inhibiting MTA1 expression at the mRNA and protein levels was verified by real-time PCR and Western blot analysis. The interaction between FOXP3 and the MTA1 promoter was verified by transcriptomic experiments. *In vitro* and *in vivo* experiments were used to determine whether the regulation of MTA1 by FOXP3 affected the invasion and migration of breast cancer cells. Immunohistochemistry was adopted to explore the correlation between the expression levels of FOXP3 and MTA1 in breast cancer samples.

**Results:**

Bioinformatics-based sequencing suggested that MTA1 is a potential downstream molecule of FOXP3. FOXP3 downregulated the expression of MTA1 in breast cancer cells by directly inhibiting MTA1 promoter activity. Importantly, FOXP3’s regulation of MTA1 affected the ability of breast cancer cells to invade and metastasize *in vitro* and *in vivo*. Moreover, analysis of clinical specimens showed a significant negative correlation between the expression levels of FOXP3 and MTA1 in breast cancer.

**Conclusion:**

We systematically explored a new mechanism by which FOXP3 inhibits breast cancer metastasis *via* the FOXP3-MTA1 pathway.

## Background

Breast cancer is a malignant tumour with a high incidence, and it is the predominant cause of cancer-related death among females (1, 2). An abundance of data show that the metastasis of tumour cells is an important cause of poor prognosis and death in breast cancer patients (3, 4). The metastasis of breast cancer is a biological process with multiple steps, and many genes and molecules can participate in its regulation (5, 6). Therefore, an in-depth study of the molecular and signal transduction pathways that play key roles in the metastasis of breast cancer is of great theoretical and clinical importance for exploring the mechanism of breast cancer metastasis.

FOXP3 (forkhead box P3), a member of the forkhead/pterygoid transcription factor family, was once considered a molecular marker of Treg cells (7, 8). In recent years, a large number of studies have shown that FOXP3 is also a tumour suppressor gene in breast cancer (9, 10). FOXP3 can regulate the expression of proto-oncogenes and tumour suppressor genes to perform its anticancer function. For instance, FOXP3 can inhibit the transcription of the proto-oncogene SKP2 (S-phase kinase-associated protein 2) and the expression of VEGF (vascular endothelial growth factor) or promote the expression of P21 (11–14). However, the mechanisms by which FOXP3 inhibits metastasis have not been fully illustrated. Thus, the molecular mechanism underlying this process needs more specific and more comprehensive study.

MTA1 (metastasis-associated 1) is one of the members of the metastasis-associated protein family and is mainly involved in regulating downstream gene transcription activity as a master regulator (15, 16). MTA1 was found to be significantly associated with local invasion and lymph node metastasis (17, 18). Nevertheless, relatively few upstream signalling pathways that regulate MTA1 have been identified.

Taken together, our findings systematically confirmed that FOXP3 could inhibit breast cancer metastasis by regulating MTA1 expression, providing an experimental basis and new ideas for the treatment of metastatic breast cancer.

## Methods

### Cell Lines and Culture

The human embryonic kidney cell line HEK293T and the breast cancer cell lines MDA-MB-231 and MCF-7 were obtained from the Type Culture Collection of the Chinese Academy of Sciences (Shanghai, China) and cultured in their corresponding media supplemented with 10% foetal bovine serum (FBS) and 100 µg/ml ampicillin/streptomycin at 37°C in a humidified atmosphere with a recommended concentration of CO_2_.

### Quantitative Real-Time PCR

Total RNA was isolated from cultured cells with RNAiso Plus (Vazyme, Nanjing, China), and cDNA was synthesized with a PrimeScript RT Reagent Kit (Vazyme). Then, cDNA and SYBR Green Ex Taq (Vazyme) were used for real-time PCR in a Prism 7500 real-time thermocycler (Applied Biosystems, Foster City, CA, USA) according to the manufacturer’s instructions. Each group was analysed in triplicate.

### Western Blot Analysis

As previously described (19), samples were separated by SDS-PAGE, transferred onto polyvinylidene fluoride (PVDF) membranes and reacted with primary antibodies against FOXP3 (10494-1-AP, Proteintech, 1:500 dilution), GAPDH (10494-1-AP, Proteintech, 1:2000 dilution) or MTA1 (#5647, CST, 1:1000 dilution) and a secondary HRP-conjugated IgG antibody. Enhanced chemiluminescence (Thermo Fisher, Waltham, MA, USA) for HRP detection was used to visualize immunoreactive protein bands.

### Construction of Stable Cell Lines

The FOXP3-overexpressing and MTA1-overexpressing lentivirus was provided by GeneChem (Shanghai, China). A stable cell line overexpressing FOXP3 (MDA-MB-231-LUC2-FOXP3), MDA-MB-231 cells stably overexpressing FOXP3 and MTA1 (MDA-MB-LUC2-FOXP3-MTA1) and the corresponding negative control cell line (MDA-MB-231-LUC2-Vector) were established using the lentivirus. Cells were treated with 2 µg/ml puromycin (Thermo Fisher, USA) to allow the optimal selection of subclones.

### Clinical Specimens and Immunohistochemistry

A total of 37 normal breast and 184 breast cancer tissue specimens were obtained from the Department of Pathology, The First Affiliated Hospital of the Fourth Military Medical University (FMMU, Shaanxi, China). Clinical staging of breast cancer was performed based on the American Joint Committee on Cancer (AJCC) staging system. Clinicopathological data were obtained from the patients’ medical records. This study was approved by our institutional research ethics committee. All specimens were immunostained for FOXP3 and MTA1 as previously described using an antibody against FOXP3 (ab10901, Abcam, 1:200 dilution) or MTA1 (#5647, CST, 1:50 dilution) (20). Sections of stained breast cancer tissue were sent to Google Biology for observation and scanning.

### Animal Studies/Tail Vein Injection Assay

All animal experiments were performed in accordance with a protocol approved by the Institutional Animal Care and Use Committee of the Fourth Military Medical University. Briefly, BALB/c nude mice (four mice per group) were injected with 100 μL of viable tumour cells (5×10^6^ cells/mL) *via* the tail vein. Successful injection was confirmed by immediate luciferase imaging. For luciferase imaging, mice were anaesthetized and injected i.p. with luciferin (25 mg/ml in 0.1 ml of phosphate-buffered saline). Fifteen minutes after injection, images were acquired using a bioluminescence imager (Caliper, USA).

### Chromatin Immunoprecipitation Assay

Chromatin immunoprecipitation (ChIP) assays were performed using an EZ ChIP Chromatin Immunoprecipitation Kit (Millipore, Billerica, USA). Briefly, pcDNA3.1-FOXP3-transfected MDA-MB-231 cells were fixed with 1% paraformaldehyde and sonicated. Then, the chromatin associated with FOXP3 was pulled down using an anti-FOXP3 antibody or control human IgG. The amounts of the specific DNA fragments were then quantified by real-time PCR and normalized to the amount of genomic DNA prepared from the same cells. Each group was analysed in triplicate.

### Statistical Analysis

Statistical analyses were performed using SPSS statistical software (SPSS 16.0, Chicago, IL, USA). The difference in FOXP3 or MTA1 expression in patients with breast cancer stratified by different clinicopathological parameters was evaluated by the Kruskal–Wallis test. Spearman rank correlation analysis was used to determine the correlation between FOXP3 expression and MTA1 immunoreactivity. Other quantitative data are presented as the mean ± standard error of mean (s.e.m.) of at least three independent experiments. Statistical significance was analysed by Student’s t test and is expressed as the P value. A p value less than 0.05 was considered statistically significant. Statistical tests were two-sided; ns, P > 0.05; *P < 0.05; **P < 0.01; and ***P < 0.001. A random number table was used to randomize the mice into the control and treatment groups.

## Results

### MTA1 Is a Potential Downstream Molecule of FOXP3

**Figure 1:**
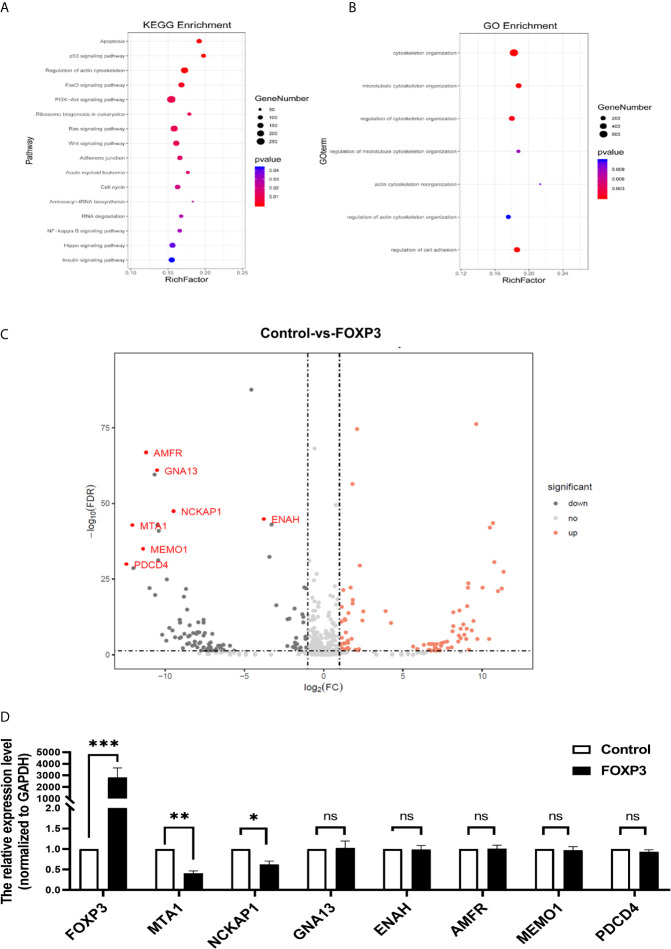
MTA1 is a downstream molecule of FOXP3. **(A)** KEGG analysis showed that the target genes of FOXP3 might be involved in the regulation of cell movement and migration pathways. **(B)** GO analysis showed that the identified differentially expressed genes participate in cell adhesion and cytoskeletal reorganization. **(C)** Seven molecules were selected from the transcriptome sequencing results. **(D)** The mRNA expression levels of these 7 potential downstream molecules were determined by real-time PCR. The data are shown as the mean ± s.e.m. ns, P > 0.05; *P < 0.05; **P < 0.01; and ***P < 0.001.

In breast cancer, FOXP3 can perform its anticancer function by regulating the expression of tumour-related genes (9). To fully understand the inhibitory effect of FOXP3 on breast cancer metastasis, high-throughput transcriptome sequencing and differential gene expression analysis were performed on MCF7-vector cells and MCF7-FOXP3 cells. We used the Kyoto Encyclopedia of Genes and Genomes (KEGG) database to identify the signalling pathways enriched with the target genes of FOXP3, and the results showed that the target genes of FOXP3 might be involved in the regulation of cell movement and migration pathways (**Figure 1A**). The differentially expressed genes identified in MCF7-vector and MCF7-FOXP3 cells were further subjected to Gene Ontology (GO) analysis to identify their participation in cellular biological processes, and the results showed that the identified differentially expressed genes participate in cell adhesion and cytoskeletal reorganization (**Figure 1B**).

Thus, we selected 7 molecules (**Figure 1C**) from the transcriptome sequencing results involved in cell adhesion, cytoskeletal reorganization, and other transfer-related pathways. We transfected the pcDNA3.1(-)-FOXP3 plasmid into MCF-7 breast cancer cells and determined the mRNA expression levels of the 7 selected potential downstream molecules of FOXP3 through real-time PCR. The results showed that after the overexpression of FOXP3 in MCF-7 cells, the mRNA expression levels of MTA1 and NCKAP1 were significantly decreased (**Figure 1D**), suggesting that they are potential downstream molecules of FOXP3. Moreover, after the overexpression of FOXP3 in MCF-7 cells, the expression level of MTA1 was significantly decreased; thus, we selected MTA1 as a potential downstream molecule of FOXP3 for subsequent experiments.

### FOXP3 Downregulates the Expression of MTA1 in Breast Cancer Cells

**Figure 2:**
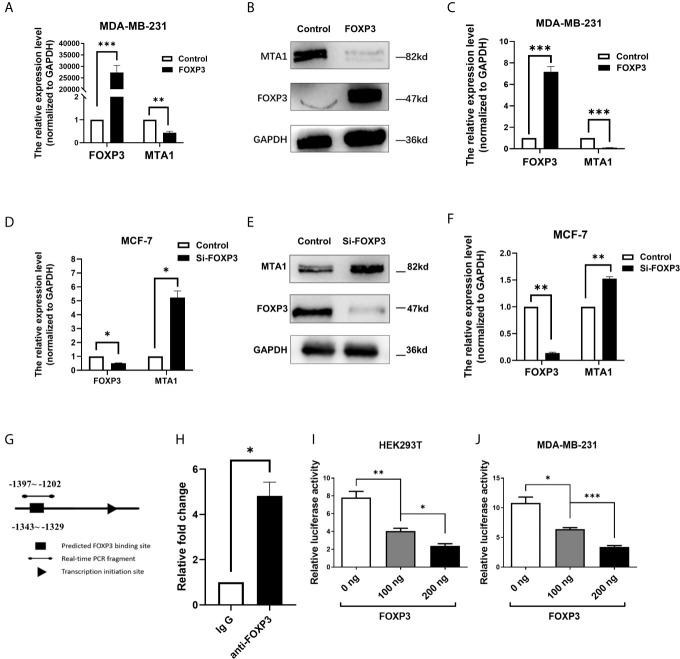
FOXP3 binds to the MTA1 promoter and reduces its expression. Real-time PCR was performed to determine the mRNA levels of FOXP3 and MTA1 in MDA-MB-231 cells **(A)** and the mRNA levels of FOXP3 and MTA1 in MCF-7 cells **(D)** in the control group and FOXP3 group. Western blotting was used to determine the protein expression levels of FOXP3 and MTA1 in MDA-MB-231 cells **(B)** and MCF-7 cells **(E)** in the control group and FOXP3 group. **(C, F)** Grey-level analysis of the bands in **(B, E)** respectively. **(G)** Potential FOXP3 binding sites in the promoter region of the MTA1 gene. **(H)** The ChIP assay showed that FOXP3 had a stronger ability to bind to specific sequences in the MTA1 promoter than to the negative IgG control. The dual-luciferase reporter assay showed that transfection with the FOXP3 overexpression plasmid significantly reduced the transcriptional activity of the MTA1 gene promoter in HEK293T cells **(I)** and MDA-MB-231 cells **(J)** in a dose-dependent manner. The data are shown as the mean ± s.e.m. ns, P > 0.05; *P < 0.05; **P < 0.01; and ***P < 0.001.

To confirm the regulation of MTA1 expression by FOXP3, MDA-MB-231 cells with low FOXP3 expression and MCF-7 cells with high FOXP3 expression were selected for the experiment according to our previous study (21). The pcDNA3.1(-)-FOXP3 plasmid was transfected into MDA-MB-231 cells. The real-time PCR (**Figure 2A**) and Western blot (**Figures 2B, C**) results showed that after the overexpression of FOXP3, the mRNA and protein expression levels of MTA1 were significantly decreased. These results verified the inhibitory effect of FOXP3 on MTA1 mRNA and protein expression.

On the other hand, Si-FOXP3 was transfected into MCF-7 cells to interfere with endogenous FOXP3 expression. The results of real-time PCR and Western blot analysis showed that Si-FOXP3 effectively reduced the expression level of FOXP3 in MCF-7 cells; in addition, with the decrease in the expression level of FOXP3 in cells, the mRNA and protein expression levels of MTA1 were significantly increased (**Figures 2D–F**). The above results further verified the inhibitory effect of FOXP3 on MTA1 expression.

### FOXP3 Binds to the MTA1 Promoter and Reduces Its Transcriptional Activity

As a transcription factor, FOXP3 often plays a regulatory role by directly binding to regulatory sequences of genes. Therefore, we analysed potential FOXP3 binding sites in the promoter region of the MTA1 gene (**Figure 2G**). Thus, we designed primers based on these prediction results. The ChIP results showed that FOXP3 had a stronger ability to bind to specific sequences in the MTA1 gene promoter than the negative control (**Figure 2H**).

To further explore whether FOXP3 can regulate gene expression after binding to the promoter of the MTA1 gene, we constructed a reporter plasmid containing the promoter of the MTA1 gene and conducted a dual-luciferase reporter assay. The results showed that in HEK293T cells, compared with transfection with the negative control plasmid, transfection with the FOXP3 overexpression plasmid significantly reduced the transcriptional activity of the MTA1 gene promoter, confirming that FOXP3 had a dose-dependent inhibitory effect on transcription driven by the MTA1 gene promoter (**Figure 2I**). The same results were observed in MDA-MB-231 cells (**Figure 2J**). These results suggest that FOXP3 can reduce the transcriptional activity of the MTA1 gene promoter and thus inhibit MTA1 expression.

### The FOXP3-MTA1 Pathway Regulates the Invasion and Migration of Breast Cancer Cells *In Vitro*

**Figure 3:**
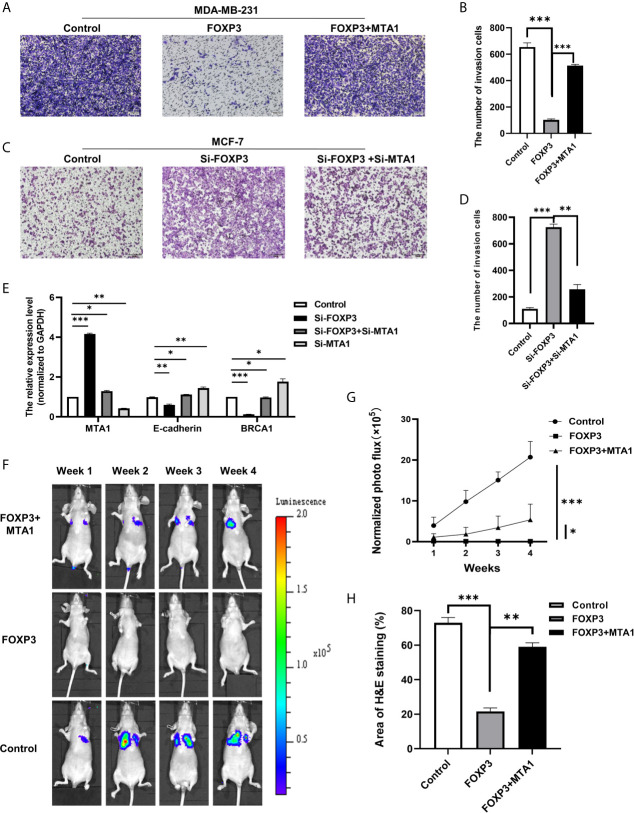
The FOXP3-MTA1 pathway regulates the invasion and migration of breast cancer cells *in vitro* and *in vivo*. Transwell assays were used to examine the invasion abilities of MDA-MB-231 cells **(A)** and MCF-7 cells **(C)**. **(B, D)** Statistical analysis of the experimental data in **(A, C)** respectively. **(E)** The mRNA expression levels of potential downstream molecules were determined. **(F)**, *In vivo* imaging technology was used to demonstrate that FOXP3 can affect the ability of breast cancer cells to metastasize *in vivo* by regulating MTA1. **(G)** Statistical analysis of fluorescence values in **(F)**. **(H)** H&E staining showed that FOXP3 inhibited lung metastasis from breast cancer by regulating MTA1 expression. The data are shown as the mean ± s.e.m. ns, P > 0.05; *P < 0.05; **P < 0.01; and ***P < 0.001.

To verify whether the regulation of MTA1 expression by FOXP3 affects the ability of breast cancer cells to metastasize *in vitro*, MDA-MB-231 cells with FOXP3 overexpression alone or in combination with MTA1 overexpression were generated, and changes in the cell invasion and migration abilities were observed through Transwell and wound healing assays. Results from the Transwell assay showed that overexpressing FOXP3 alone significantly decreased the migration abilities of MDA-MB-231 cells, and importantly, the ability of FOXP3 to inhibit invasion was reversed with MTA1 overexpression (**Figures 3A, B**). The same results were observed in the wound healing assay (**Supplementary Figure 1A, C**).

We also generated MCF-7 cells in which FOXP3 alone was silenced or FOXP3 was silenced in combination with MTA1 and evaluated changes in cell invasion and migration abilities. Results from the Transwell assay showed that compared with negative control cells, MCF-7 cells in which FOXP3 was silenced had significantly increased invasion abilities. In addition, compared with MCF-7 cells in which FOXP3 alone was silenced, MCF-7 cells in which both FOXP3 and MTA1 were silenced had significantly decreased invasion abilities (**Figures 3C, D**). The same results were observed in the wound healing assay (**Supplementary Figures 1B, D**). These results demonstrate that FOXP3’s regulation of MTA1 expression affects the ability of breast cancer cells to invade and migrate *in vitro*.

To determine the downstream molecules of the FOXP3-MTA1 pathway, we selected molecules downstream of MTA1 according to related studies. We transfected Si-FOXP3 and Si-MTA1 into MCF-7 breast cancer cells and determined the mRNA expression levels of the selected potential downstream molecules of MTA1 through real-time PCR. The results showed that with the decrease in MTA1 after transfection with Si-FOXP3 or transfection with both Si-FOXP3 and Si-MTA1, the mRNA expression levels of E-cadherin and BRCA1 were significantly increased, suggesting that they are potential downstream molecules of the FOXP3-MTA1 pathway (**Figure 3E**).

### The FOXP3-MTA1 Pathway Regulates Lung Metastasis From Breast Cancer Cells *In Vivo*

To evaluate the effect of FOXP3 on the expression of MTA1 and on breast cancer metastasis *in vivo*, a lentiviral infection strategy was used to generate MDA-MB-231 cells overexpressing FOXP3 (MDA-MB-231-LUC2-FOXP3), MDA-MB-231 cells stably overexpressing FOXP3 and MTA1 (MDA-MB-LUC2-FOXP3-MTA1) and the corresponding negative control cells (MDA-MB-231-LUC2-Vector). The real-time PCR results indicated that all three stable cell lines were successfully constructed (**Supplementary Figures 2A, B**). In addition, the generated cells in a specific number of wells from a 96-well plate were imaged, and the results showed a linear correlation between luciferase activity and the number of cells (**Supplementary Figures 2C, D**).

We injected the generated stable cell lines into nude mice through the caudal vein and monitored the ability of breast cancer cells to metastasize to the lungs *via in vivo* imaging technology. Compared with those of mice inoculated with MDA-MB-231-LUC2-Vector cells, no significant bioluminescence signals were determined in the lungs of nude mice 28 days after inoculation with MDA-MB-231-LUC2-FOXP3 cells. However, compared with that of MDA-MB-231-LUC2-FOXP3 cells, the lung metastatic ability of MDA-MB-LUC2-FOXP3-MTA1 cells was restored to a certain extent (**Figures 3F, G**). These results demonstrate that FOXP3 can affect the ability of breast cancer cells to metastasize *in vivo* by regulating MTA1 expression. Then, we removed lung tissues from nude mice and further identified lung metastases of breast cancer by H&E staining (**Supplementary Figure 3A**). The results showed that mice in both the negative control group and the FOXP3-MTA1 group had breast cancer metastasis to the lungs, while mice in the FOXP3 group did not show significant breast cancer metastasis to the lungs (**Figure 3H**), confirming that FOXP3 can inhibit the metastasis of breast cancer cells by regulating MTA1 expression *in vivo*.

### FOXP3 Expression and MTA1 Expression Are Negatively Correlated in Clinical Breast Cancer Samples

**Figure 4:**
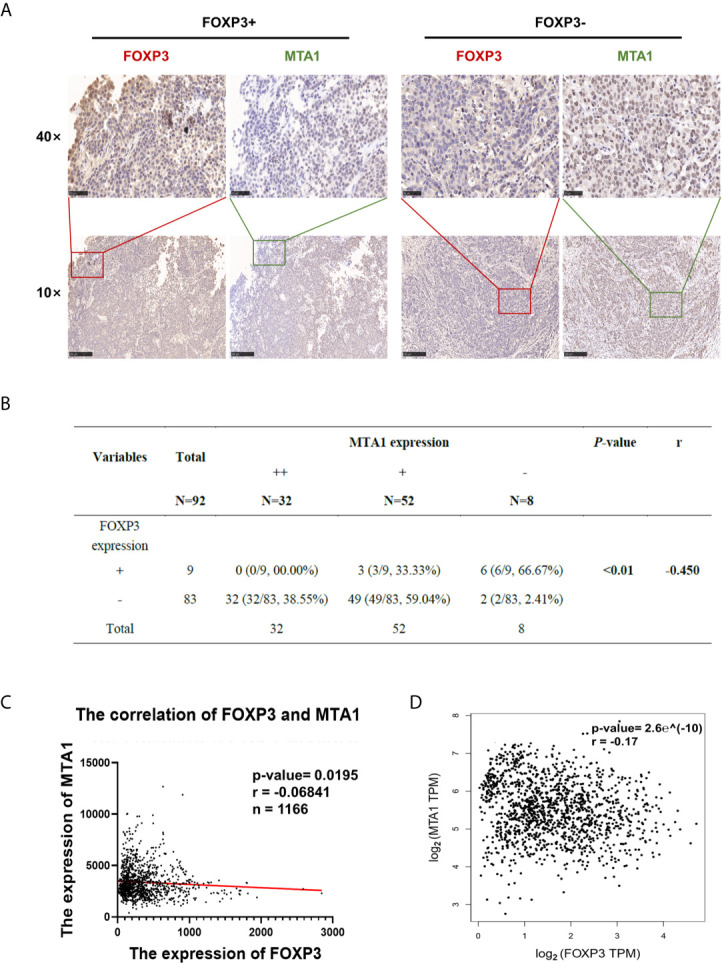
FOXP3 expression and MTA1 expression were negatively correlated in clinical breast cancer samples. **(A)** The expression level of MTA1 was lower in breast cancer tissues with positive FOXP3 expression than in breast cancer tissues with negative FOXP3 expression. FOXP3 expression was negatively correlated with MTA1 expression. Scale bars, 250 μm (10×) and 50 μm (40×). **(B)** A negative correlation between nuclear FOXP3 expression and MTA1 expression was found in breast cancer specimens. Spearman rank correlation analysis. **(C)** Analysis of 1166 breast cancer patients in the TCGA database showed a negative correlation between FOXP3 and MTA1 expression levels in clinical breast cancer samples. Pearson correlation analysis. **(D)** Data from breast cancer cases were analysed on the GEPIA website, verifying that the expression levels of FOXP3 and MTA1 were negatively correlated in clinical breast cancer samples. Pearson correlation analysis.

To explore the clinical significance of FOXP3 and MTA1 expression in the development of breast cancer, we evaluated FOXP3 and MTA1 expression in 92 breast cancer tissues. We defined FOXP3 positivity and MTA1 positivity as nuclear staining only and classified the expression level of MTA1 into three grades (**Supplementary Figures 3B, C**). We analysed the correlations between lymph node metastasis from breast cancer, pathological stage and the expression levels of FOXP3 and MTA1. The results showed that the expression level of FOXP3 in the nucleus was negatively correlated with lymph node metastasis from breast cancer and pathological stage, while the expression level of MTA1 was positively correlated with these parameters (**Figure 4A**). Furthermore, we analysed the correlation between FOXP3 and MTA1 expression in 92 breast cancer tissues. The results showed that the expression level of MTA1 was negatively correlated with the expression level of FOXP3 (P < 0.01) (**Figure 4B**).

To further verify the correlation between FOXP3 and MTA1 expression in breast cancer, we downloaded and analysed breast cancer case data from public databases. Analysis of 1166 breast cancer patients in The Cancer Genome Atlas (TCGA) database and breast cancer case data in GEPIA showed that the FOXP3 expression level was negatively correlated with the MTA1 expression level (**Figures 4C, D**), consistent with our results.

## Discussion

In breast cancer, FOXP3 can regulate the expression of tumour-related genes to perform its anticancer function. However, the mechanism(s) by which FOXP3 inhibits metastasis have not been fully illustrated. In our attempt to better understand FOXP3’s inhibitory effect on breast cancer metastasis, we found that MTA1, which is associated with breast cancer metastasis, may be a downstream molecule of FOXP3. Then, we confirmed that FOXP3 can inhibit the expression of MTA1 by binding to the promoter of MTA1 and thus reduce the ability of breast cancer cells to metastasize. Our experiment revealed that MTA1 is a new target gene of FOXP3 that regulates breast cancer metastasis and provides further knowledge about the mechanism of FOXP3 in breast cancer metastasis.

As a transcription factor, FOXP3 often plays a regulatory role by directly binding to the regulatory sequences of genes. We found the potential binding site of FOXP3 in the promoter region of MTA1 and verified that FOXP3 can decrease the transcriptional activity of the MTA1 promoter and therefore inhibit MTA1 expression. Our results were also consistent with those of previous studies demonstrating that FOXP3 plays a regulatory role by directly binding to the promoters of downstream molecules, such as HER2, CD44 and VEGF (12, 21–23). Based on the above suggestions, we assumed that the ability of FOXP3 to enter the nucleus as a transcription factor is a requirement for it to regulate downstream gene expression. FOXP3, when trapped in the cytoplasm, fails to function properly as a transcription factor, as confirmed by previous research in our laboratory (21). Therefore, in this study, when we analysed clinical breast cancer samples, we defined samples with only nuclear staining as positive for FOXP3. On this premise, we verified that FOXP3 expression and MTA1 expression were negatively correlated in breast cancer tissues. Combined with the results of previous *in vitro* and *in vivo* experiments, these results demonstrated that FOXP3 can hinder breast cancer metastasis by downregulating MTA1 expression.

In addition, our results showed that the *in vivo* lung metastatic ability of cells stably overexpressing both FOXP3 and MTA1 was still lower than that of negative control cells. This pattern suggests that FOXP3 regulates the expression of downstream molecules in addition to MTA1 to inhibit breast cancer metastasis. A related study confirmed that FOXP3 can inhibit breast cancer metastasis by downregulating CXCR4 and CD44 (24) (21). FOXP3, as a tumour suppressor molecule in breast cancer, plays a variety of tumour suppressor roles by regulating the transcriptional levels of a series of tumour-related genes. There are still many downstream molecules associated with breast cancer metastasis in the FOXP3 regulatory network that have not yet been discovered and studied.

MTA1 is a member of the metastasis-associated family and is mainly involved in regulating the transcriptional activity of downstream genes as a coregulator. It has been determined that the MTA family has abundant downstream targets. For instance, MTA1 can affect the transcription of P53 (25, 26) and BRCA1 (27) and the protein expression of WNT1 (28) and E-cadherin (29) to promote tumour occurrence and metastasis. However, relatively little is known about the upstream regulators of MTA1. Only a few factors, such as hypoxia-inducible factor-1α (HIF1α) (30) and C-MYC (31), have been identified as inducing factors for MTA1. In this study, we identified a new upstream regulator of MTA1 and validated this new FOXP3-MTA1 regulatory pathway through *in vitro* and *in vivo* experiments, identifying a new pathway for the study of tumour metastasis. Based on the known function of MTA1, we hypothesize that the downstream signalling molecules of the FOXP3-MTA1 pathway are cytoskeletal motility-related molecules that mediate tumour metastasis, and this possibility will be explored further in our future experiments.

In summary, through *in vitro* cell assays, *in vivo* animal experiments, and analysis of clinical breast cancer samples, we systematically confirmed the regulation of MTA1 expression by FOXP3 and clarified its mechanism. Our study provides not only further knowledge about the mechanism by which FOXP3 inhibits breast cancer metastasis but also an experimental basis and new ideas for the treatment of metastatic breast cancer.

## Data Availability Statement

The datasets presented in this study can be found in online repositories. The names of the repository/repositories and accession number(s) can be found in the article/**Supplementary Material**.

## Ethics Statement

The animal study was reviewed and approved by the Institutional Animal Care and Use Committee of the Fourth Military Medical University.

## Author Contributions

All authors listed have made a substantial, direct, and intellectual contribution to the work, and approved it for publication.

## Funding

This study was supported by grants from the National Natural Science Foundation of China (NSFC) (Nos. 82072910, 81902678, 81672864, 81802632, 81702590, 81672800) and the Key Research and Development Program of Shaanxi Province (Nos. 2021SF-207, 2017ZDCXL-SF-01-03, 2019SF-087).

## Conflict of Interest

The authors declare that the research was conducted in the absence of any commercial or financial relationships that could be construed as a potential conflict of interest.
