# Supplementary material for: FOXP3 Inhibits the Metastasis of Breast Cancer by Downregulating the Expression of MTA1
Source: Front Oncol. 2021 Jul 7;11:656190. doi: 10.3389/fonc.2021.656190 (PMC8293273; doi:10.3389/fonc.2021.656190)
Supplement: Supplementary file 1 [file DataSheet_1.docx]

- **Supplementary Figures:**

**Supplementary Figure 1**

**
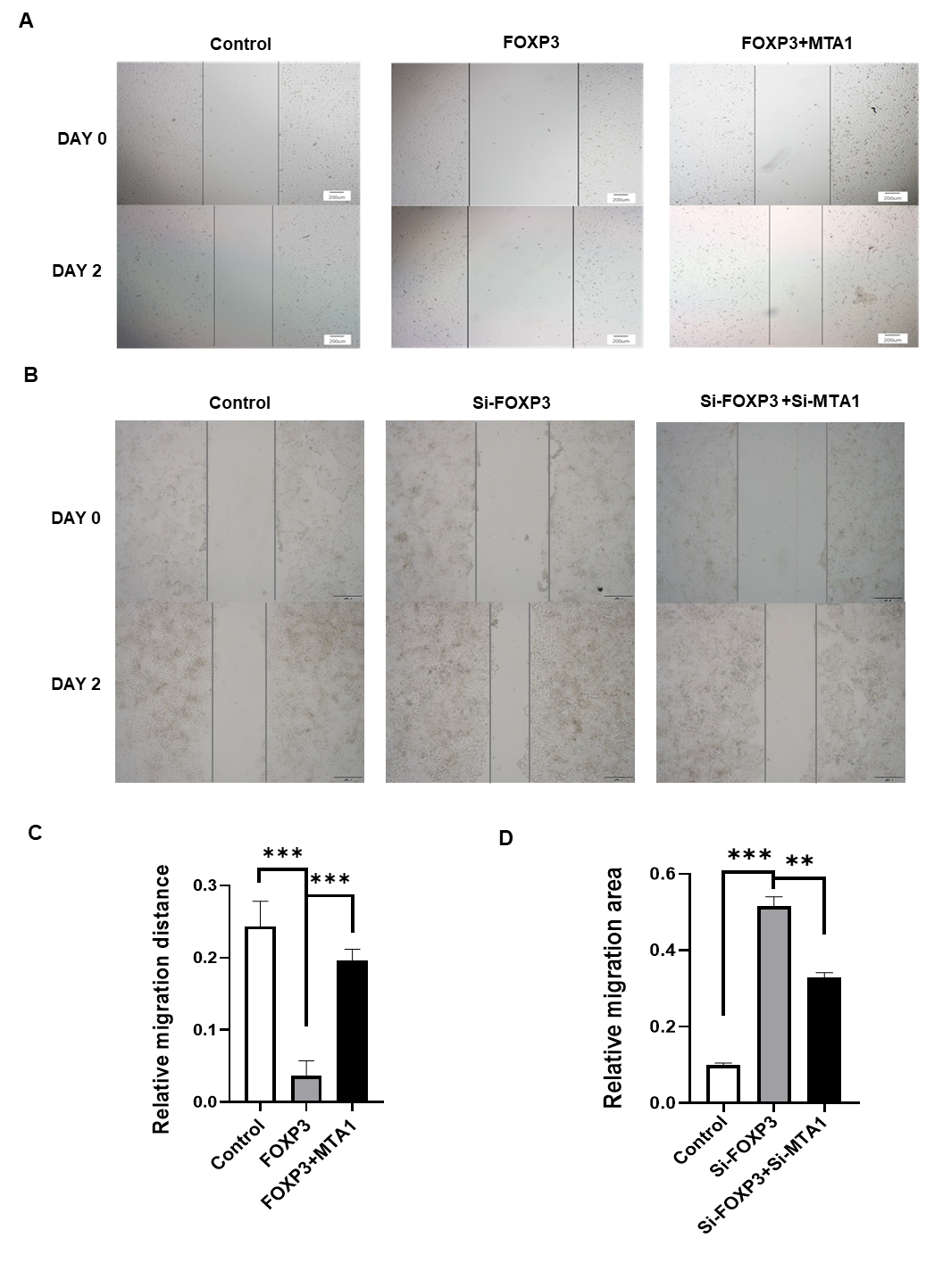
**

**Figure legend.** The FOXP3-MTA1 pathway regulates the invasion and migration of breast cancer cells in vitro. A, The cell migration assay showed that compared with negative control cells, MDA-MB-231 cells had significantly decreased invasion and migration abilities after overexpressing FOXP3. B, The cell migration assay showed that compared with MCF-7 cells in which FOXP3 alone was silenced, MCF-7 cells in which both FOXP3 and MTA1 were silenced had significantly decreased invasion and migration abilities. C and D, Statistical analysis of the experimental data in A and B, respectively.

**Supplementary Figure 2**

**
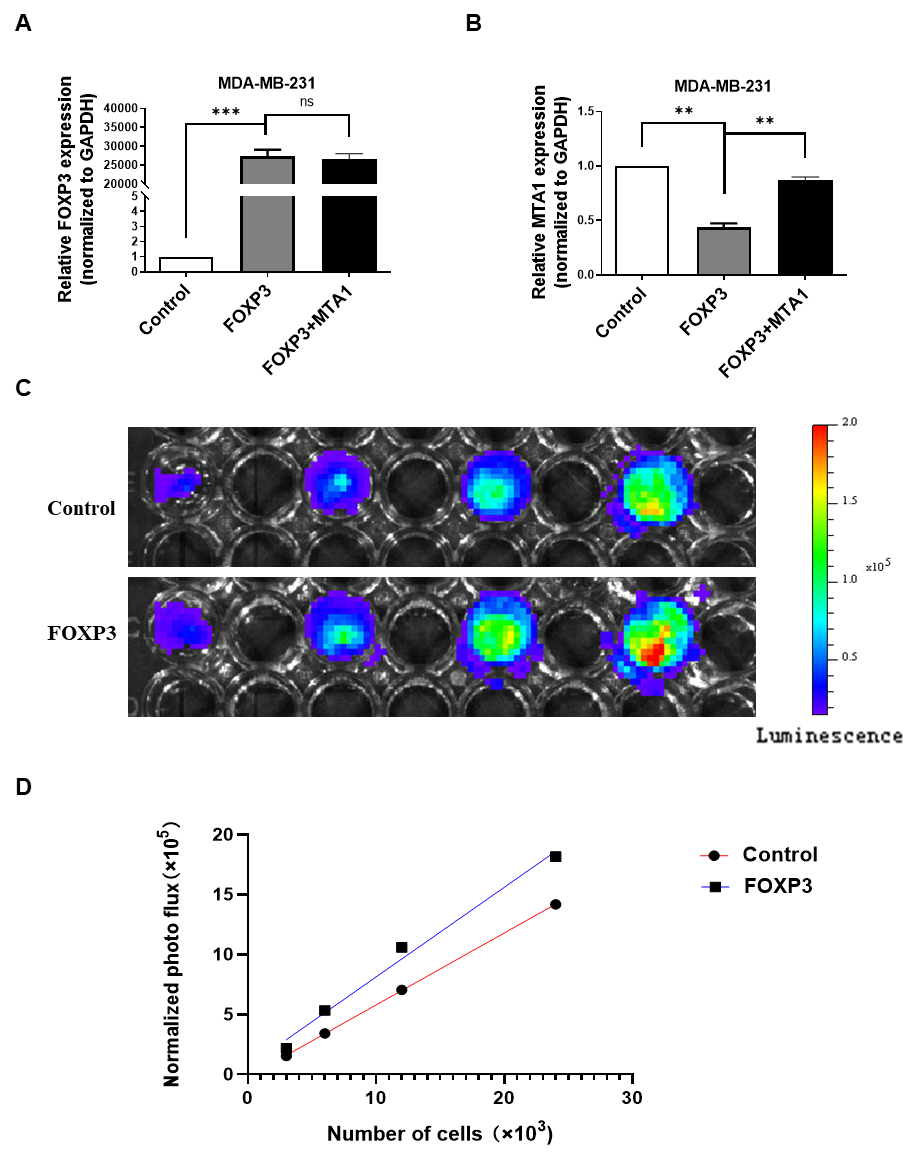
**

**Figure legend.** Cell lines stably overexpressing FOXP3 or MTA1 were successfully generated. Real-time PCR was applied to evaluate the expression of FOXP3 (A) and MTA1 (B) in the FOXP3 group and FOXP3+ MTA1 group of generated MDA-MB-231 cells and indicated that the three stable cell lines were successfully generated. C, The generated cells in a specific number of wells of a 96-well plate were imaged. D, Linear regression analysis of the fluorescence signal intensities and cell numbers, with the results showing a linear correlation between luciferase activity and the number of cells.

**Supplementary Figure 3**

**
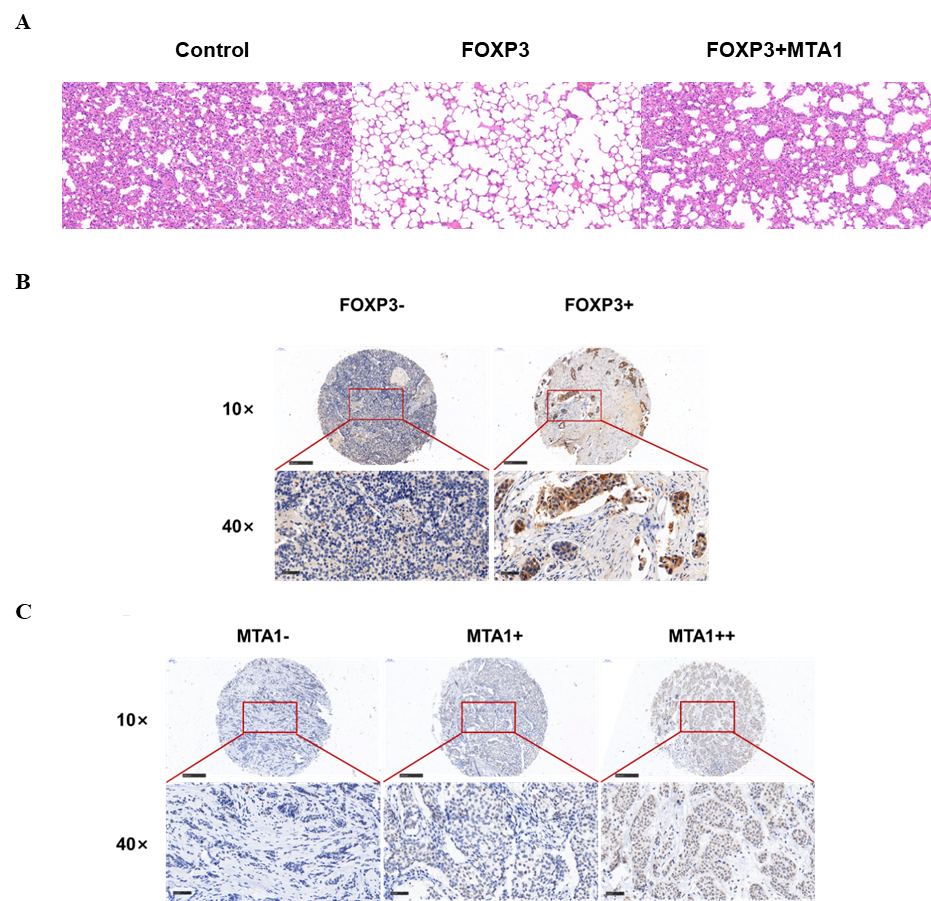
**

**Figure legend.** A**,** H&E staining showed that FOXP3 inhibited lung metastasis from breast cancer by regulating MTA1 expression. B, Representative immunohistochemical images of nuclear FOXP3 expression in breast cancer specimens. Scale bars, 250 μm (10×) and 50 μm (40×). The expression of FOXP3 was classified as positive (+) or negative (-), and we defined tissues with only nuclear staining or both nuclear and cytoplasmic staining as positive for FOXP3. C, Representative immunohistochemical images of MTA1 expression in breast cancer specimens. Scale bars, 250 μm (10×) and 50 μm (40×). The expression level of MTA1 was classified into the following three grades: -, no breast cancer cells with observable colour or < 25% of breast cancer cells exhibiting slight colour; +, <75% of breast cancer cells exhibiting colour; and ++, > 75% of breast cancer cells exhibiting colour.

**Supplementary Tables:**

**Supplementary table 1:**

Tissue microarray and immunohistochemical analyses showed that the FOXP3 expression level in the nucleus was negatively correlated with lymph node metastasis and pathological stage in breast cancer. Wilcoxon rank-sum test.

| **Variable** | **Total** | **FOXP3 expression** | | ***P*-value** |
| --- | --- | --- | --- | --- |
|  |  | **+** | **-** |  |
|  | **N=92** | **N=9** | **N=83** |  |
| No. of node  metastases |  |  |  |  |
| N0: none | 47 | 5 (5/47, 10.64%) | 42 (42/47, 89.36%) | **<0.001** |
| N1: 1-3 | 22 | 3 (3/22, 13.64%) | 19 (19/22, 86.36%) |  |
| N2: 4-9 | 10 | 1 (1/10, 10.00%) | 9 (9/10, 90.00%) |  |
| N3: ≥10 | 13 | 0 (0/13, 0.00%) | 13 (13/13, 100.00%) |  |
| Total | 92 | 9 | 83 |  |
| Clinical stage |  |  |  |  |
| AJCC Ⅰ | 3 | 1 (1/3, 33.33%) | 2 (2/3, 66.67%) | **<0.001** |
| AJCC Ⅱ | 50 | 7 (7/50, 14.00%) | 43 (43/50, 86.00%) |  |
| AJCC Ⅱ-Ⅲ | 13 | 1 (1/13, 7.69%) | 12 (12/13, 92.31%) |  |
| AJCC Ⅲ | 26 | 0 (0/26, 0.00%) | 26 (26/26, 100.00%) |  |
| Total | 92 | 9 | 83 |  |

**Supplementary table 2:**

Tissue microarray and immunohistochemical analyses showed that the MTA1 expression level was positively correlated with lymph node metastasis and pathological stage in breast cancer. Spearman rank correlation analysis.

| **Variable** | **Total** | **MTA1 expression** | | | ***P*-value** | **r** |
| --- | --- | --- | --- | --- | --- | --- |
|  |  | **++** | **++** | **-** |  |  |
|  | **N=92** | **N=32** | **N=52** | **N=8** |  |  |
| No. of node  metastases |  |  |  |  |  |  |
| N0: none | 47 | 7 (7/47, 14.89%) | 35 (35/47, 74.47%) | 5 (5/47, 10.64%) | **<0.01** | **0.478** |
| N1: 1-3 | 22 | 7 (7/22, 31.82%) | 13 (13/22, 59.09%) | 2 (2/22, 9.09%) |  |  |
| N2: 4-9 | 10 | 7 (7/10, 70.00%) | 2 (2/10, 20.00%) | 1 (1/10, 10.00%) |  |  |
| N3: ≥10 | 13 | 11 (11/13, 84.62%) | 2 (2/13, 15.38%) | 0 (0/13, 0.00%) |  |  |
| Total Clinical stage |  | 32 | 52 | 8 |  |  |
| AJCC Ⅰ | 3 | 0 (0/3, 0.00%) | 3 (3/3, 100.00%) | 0 (0/3, 0.00%) | **<0.01** | **0.446** |
| AJCC Ⅱ | 50 | 10 (10/50, 20.00%) | 33(33/50, 66.00%) | 7 (7/50, 14.00%) |  |  |
| AJCC Ⅱ-Ⅲ | 13 | 4 (4/13, 30.77%) | 8 (8/13, 61.54%) | 1 (1/13, 7.69%) |  |  |
| AJCC Ⅲ | 26 | 18 (18/26, 69.23%) | 8 (8/26, 30.77%) | 0 (0/26, 0.00%) |  |  |
| Total |  | 32 | 52 | 8 |  |  |

**Supplementary table 3:** Primers used in this study

| **Annotation** | **Primer sequence (5’-3’)** | |
| --- | --- | --- |
| FOXP3 | forward | 5' CTCTTCTTCCTTGAACCCCAT 3' |
|  | reverse | 5' CTGGAGGAGTGCCTGTAAG 3' |
| MTA1 | forward | 5' TGGAAGAAATATGGTGGCTTGA 3' |
|  | reverse | 5' TTCGTCGTGTGCAGATAGAAAG 3' |
| NCKAP1 | forward | 5' ACATCATATTGGAGATCGCAGT 3' |
|  | reverse | 5' CAGTGATGAGATTTCGAGCTTG 3' |
| GNA13 | forward | 5' GCAACGTGATCAAAGGTATGAG 3' |
|  | reverse | 5' CTCCATGTTGTTGGTTTGAGTT 3' |
| ENAH | forward | 5' CATACAGGCAACAACACATTCA 3' |
|  | reverse | 5' AAAGTTGAGACCATACACCTGT 3' |
| AMFR | forward | 5' CGAGTTTTTCGGTTGAAGTGAT 3' |
|  | reverse | 5' GAATCTGATGAGCCATTGCATT 3' |
| MEMO1 | forward | 5' ATGCAGGATATACGTACTGTGG 3' |
|  | reverse | 5' AGGTCATACAGAGGTGTCCTAT 3' |
| PDCD4 | forward | 5' ATGTCTAAAGGTGGAAAGCGTA 3' |
|  | reverse | 5' TGGTGAAAATGAGGTACTTCCA 3' |
| GAPDH | forward | 5' GCTCGTCGTCGACAACGGCTC 3' |
|  | reverse | 5' CAAACATGATCTGGGTCATCTTCTC 3' |

**Supplementary table 4:** siRNAs used in this study

| **Annotation** | siRNA **sequence** | |
| --- | --- | --- |
| FOXP3 | sense | 5' GCAGCGGACACUCAAUGAG 3' |
|  | antisense | 5' CUCUUUGUGUGUCCGCUGC 3' |
| MTA1 | sense | 5' GCAUCAUUGAGUACUACUATT 3' |
|  | antisense | 5' UAGUAGUACUCAAUGAUGCTT 3' |
| Negative control | sense | 5' UUCUCCGAACGUGUCACGUTT 3' |
|  | antisense | 5' ACGUGACACGUUCGGAGAATT 3' |
